# Autologous protein solution and 3D-Printed technologies as potential adjuncts to arthroscopic elbow release: a narrative review and conceptual framework

**DOI:** 10.3389/fbioe.2026.1883349

**Published:** 2026-09-18

**Authors:** Haoran Li, Haiyang Xing, Shaohui Zhang, Ling Zhang, Jinwen Kang

**Affiliations:** 1 Department of Sports Medicine, Cangzhou Hospital of Integrated TCM-WM HEBEI, Cangzhou, China; 2 Hebei Key Laboratory of Integrated Traditional and Western Medicine in Osteoarthrosis Research, Cangzhou, China; 3 Department of Joint Surgery, Cangzhou Hospital of Integrated TCM-WM HEBEI, Cangzhou, China

**Keywords:** arthroscopic elbow release, autologous protein solution, clinical evidence, digital orthopedics, practical considerations

## Abstract

Post-traumatic and degenerative elbow stiffness is a major cause of upper-limb disability, as relatively small losses of elbow and forearm motion can significantly impair activities of daily living and work capacity. Arthroscopic elbow release has evolved as a joint-preserving alternative to open arthrolysis in selected cases. Multiple series have demonstrated meaningful gains in flexion including extension arc and forearm rotation, high patient satisfaction, and low complication rates. However, residual pain, recurrent stiffness, and progressive osteoarthritic change remain problematic in a subset of patients. In parallel, autologous protein solution (APS), an autologous blood-derived product enriched in anti-inflammatory cytokines and anabolic factors, has shown promising preclinical effects in modulating IL-1/TNF-driven cartilage catabolism. Clinical studies in knee osteoarthritis report that single intra-articular APS injections are safe and can improve pain and function in selected patients with mild-to-moderate disease, although recent randomized data are mixed and benefits appear attenuated in advanced disease. Importantly, evidence for APS in upper-limb joints, particularly the elbow, remains essentially unavailable. Three-dimensional (3D) printed digital orthopedics offers expanded options for preoperative planning and personalized bracing in complex elbow conditions, though high-quality clinical trials are lacking. This review synthesizes current evidence on APS in osteoarthritis, arthroscopic elbow release, and 3D-printed digital orthopedics, proposing a hypothetical joint-preserving pathway where arthroscopic release restores mechanics, APS provides biological modulation in selected patients, and 3D-printed devices personalize planning and postoperative rehabilitation. We emphasize that this integrated approach is currently based on indirect evidence and requires formal clinical validation before adoption into practice.

## Introduction

Post-traumatic and degenerative elbow stiffness is a major cause of upper-limb disability, because relatively small losses in elbow and forearm motion can markedly impair activities of daily living and work capacity (Fan et al., 2025; Akhtar et al., 2021). Classical studies suggested that a flexion-extension arc of 30°–130° and 50° of forearm rotation are sufficient for basic tasks (Vasen et al., 1995), yet more recent analyses indicate that modern daily activities may demand a broader functional range of motion (Gates et al., 2016). As a result, patients with stiff elbows often experience substantial functional and quality-of-life limitations (Fan et al., 2025).

Arthroscopic elbow release has evolved as a joint-preserving alternative to open arthrolysis in selected cases of post-traumatic and degenerative stiffness (Beck et al., 2022). Several studies consistently report meaningful gains in flexion-extension and forearm rotation, with high rates of pain relief and patient satisfaction when procedures are performed by experienced surgeons (Beck et al., 2022; Roth et al., 2026). However, residual pain, recurrent stiffness and progressive osteoarthritic changes remain important challenges in a subset of patients, highlighting the need for strategies that address both mechanical impingement and the underlying inflammatory–degenerative environment (Beck et al., 2022). In this context, orthobiologic therapies have attracted increasing interest as adjuncts for joint preservation.

Autologous protein solution (APS) is an autologous blood-derived product enriched in anti-inflammatory cytokines and anabolic factors, developed to modulate intra-articular inflammation and cartilage catabolism (Bin Abd Razak et al., 2021). APS is typically prepared using the nSTRIDE system, which processes approximately 60 mL of autologous blood to produce 3–4 mL of final product for intra-articular injection. Early clinical data in knee osteoarthritis suggest that APS injections can improve pain and function in some patient populations, although recent randomized trials have yielded mixed results and the overall magnitude and durability of benefit remain uncertain (Kuwasawa et al., 2023; Gupta, 2024; Ross et al., 2024). Evidence for APS in upper-limb joints is emerging but still extremely limited, and data specific to the elbow are essentially absent.

At the same time, three-dimensional (3D)-printed digital orthopedics has expanded the possibilities for preoperative planning and personalized postoperative support in complex elbow conditions (Grunert et al., 2023; Huszar et al., 2024). 3D-printed anatomical models and patient-specific instruments can enhance visualization and accuracy in managing post-traumatic deformity and stiffness, while custom 3D-printed orthoses and fixation devices enable tailored motion control and improved comfort during rehabilitation (Grunert et al., 2023). However, most evidence consists of case reports and small case series, with no randomized controlled trials available. Against this background, integrating APS with arthroscopic elbow release and 3D-printed digital orthopedics represents a hypothetical joint-preserving concept that has not been clinically tested. This narrative review has two aims. The primary aim is to synthesize current evidence on APS in osteoarthritis and upper-limb conditions, to summarize the outcomes of arthroscopic elbow release, and to review 3D-printed applications around the elbow. The secondary aim is to use this evidence to outline a conceptual, hypothesis-generating pathway for the combined use of these three approaches and to define priorities for future research.

## Methods

This is a narrative review, not a systematic review, and it is not registered in PROSPERO. Consistent with the Scale for the Assessment of Narrative Review Articles (SANRA) framework for non-systematic reviews (Baethge et al., 2019), we searched PubMed, Scopus, and Web of science from database inception to 13 April 2026 using combinations of the terms “autologous protein solution,” “APS,” “elbow stiffness,” “post-traumatic elbow,” “arthroscopic elbow release,” “arthrolysis,” “elbow osteoarthritis,” “3D printing,” “3D-printed orthosis,” and “patient-specific instrumentation.” Reference lists of retrieved articles and recent systematic reviews were hand-searched for additional relevant studies. English-language clinical studies, systematic reviews, and preclinical/mechanistic studies relevant to APS, arthroscopic elbow release, or 3D-printed orthopedic applications around the elbow were considered for inclusion; conference abstracts without peer-reviewed full text and non-English publications were excluded. Given the narrow and heterogeneous nature of the elbow-specific literature, priority was given to systematic reviews and randomized controlled trials where available, supplemented by case series and case reports when higher-level evidence was absent, as explicitly noted throughout the text. Selection and synthesis of the included literature were performed by the authors through iterative review and discussion rather than a formal dual-reviewer screening process, consistent with standard narrative review methodology. No formal risk-of-bias or GRADE certainty assessment was performed; instead, the directness and level of each body of evidence (elbow-specific clinical, indirect clinical from other joints, preclinical, or purely conceptual) are explicitly flagged where relevant throughout the manuscript.

### Biologic rationale of APS for joint preservation

Osteoarthritis and post-traumatic joint degeneration are driven by a chronic imbalance between pro- and anti-inflammatory mediators in synovial fluid, with interleukin one and tumor necrosis factor alpha playing central roles in stimulating matrix-degrading enzymes and progressive cartilage loss (Punzi et al., 2016). APS is an autologous blood-derived product formulated by processing leukocyte-rich platelet-rich plasma over specialized beads to concentrate anti-inflammatory cytokines (notably interleukin-1 receptor antagonist, soluble TNF receptors, and IL-10) and anabolic growth factors, while relatively lowering levels of catabolic cytokines. This composite profile is intended to simultaneously block IL-1- and TNF-mediated signaling and support chondrocyte survival and matrix synthesis (Bin Abd Razak et al., 2021; O'Shaughnessey et al., 2014).

In practice, APS is generated through a two-step, point-of-care centrifugation process: whole blood is first processed in a cell separator to isolate leukocyte-rich platelet-rich plasma (typically ∼55–60 mL of blood centrifuged at approximately 3200 rpm for 15 min to yield ∼6 mL of PRP), which is then passed through a second concentrator containing polyacrylamide beads and centrifuged again (approximately 2000 rpm for 2 min) to produce 2–4 mL of final APS product (van Drumpt et al., 2016; Gupta and Maffulli, 2025). This process typically yields white blood cell concentrations approximately 11-fold and platelet concentrations approximately 3-fold above whole-blood baseline, although concentration factors and the resulting cytokine and growth factor profile can vary meaningfully between individual preparations and patients (van Drumpt et al., 2016). This inherent product heterogeneity is a recognized source of variability that may contribute to inconsistent clinical outcomes across studies and should be considered when interpreting or comparing results between trials.

Preclinical work has provided mechanistic support for this concept, so that in bovine cartilage explant models exposed to IL-1α or TNFα, APS reduced glycosaminoglycan loss, preserved Safranin-O staining, and completely inhibited IL-1α-induced collagen release over 21 days, outperforming recombinant IL-1Ra and soluble TNF-RI used alone (Matuska et al., 2013). Studies on human chondrocytes have shown that APS suppresses production of matrix metalloproteinase-13 and other catabolic mediators in response to IL-1β and TNFα, indicating effective interruption of key degradative pathways (Woodell-May et al., 2011). In a randomized, controlled rat medial meniscal-tear model of post-traumatic osteoarthritis, a single intra-articular injection of human APS significantly reduced collagen and cartilage degeneration and improved the total histologic joint score compared with saline controls 3 weeks after treatment, providing direct *in vivo* evidence of a disease-modifying effect (King et al., 2017).

From a joint-preservation perspective, these data support the use of APS as a biological adjunct in settings where mechanical factors have been optimized but an inflammatory-degenerative environment persists. In the context of arthroscopic elbow release, capsular release and osteophyte debridement can restore motion and alleviate impingement, yet residual synovitis and cartilage damage may continue to drive pain and structural progression (Merolla et al., 2015; Ravalli et al., 2019). However, extrapolating APS effects from the knee to the elbow warrants caution, as the two joints differ substantially in anatomy and biomechanics. The elbow is a non-weight-bearing, highly congruent trochoginglymoid joint whose stability derives predominantly from its bony architecture and collateral ligaments, whereas the knee is a weight-bearing, meniscus-containing joint subjected to much greater cyclical loads; post-traumatic elbow stiffness is frequently driven more by capsular contracture, heterotopic ossification, and intra-articular fibrosis than by primary cartilage loss (Merolla et al., 2015; Ravalli et al., 2019). The elbow’s smaller joint volume and synovial surface area may also alter the effective concentration and residence time of an intra-articularly injected product such as APS relative to the knee, for which current dosing (typically 3–4 mL) has been calibrated. These anatomical and biomechanical differences have not been empirically evaluated for APS and should be regarded as open questions rather than established modifiers of treatment response. Conceptually, combining arthroscopic correction of mechanics with intra articular APS could help dampen postoperative inflammatory cascades, protect remaining cartilage, and potentially slow osteoarthritic progression, extrapolating from the knee and preclinical animal literature (Bin Abd Razak et al., 2021; King et al., 2017). Although this rationale has not been directly tested in the elbow, it provides a biologically coherent framework for considering APS as part of a joint preserving strategy around arthroscopic elbow surgery (Figure 1).

**FIGURE 1:**
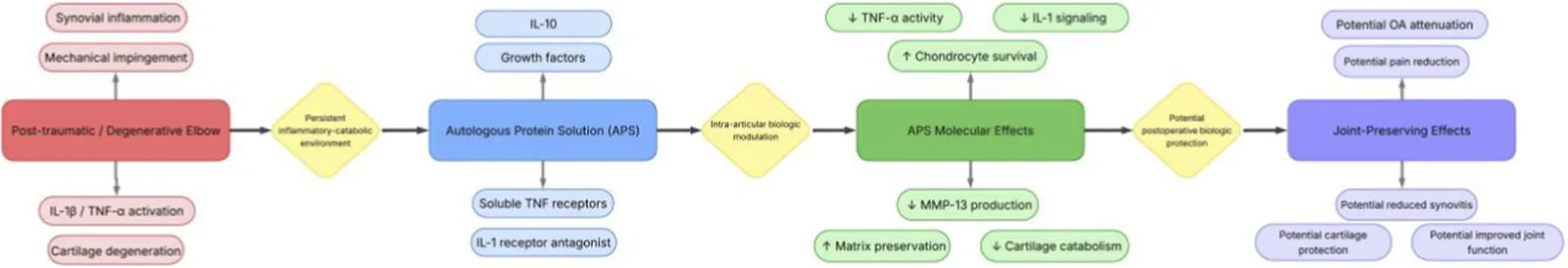
Conceptual schematic of the biologic rationale for autologous protein solution (APS) in the post-traumatic or degenerative elbow, illustrating the hypothesized pathway from persistent inflammatory–catabolic activity (IL-1β/TNF-α) through APS-mediated cytokine modulation to potential joint-preserving effects. Effects labeled “potential” are extrapolated from knee and preclinical data and have not been demonstrated in the elbow.

### Clinical evidence of APS in osteoarthritis and upper-limb conditions

In a recent scoping review, Gupta summarized six clinical studies of intra-articular APS for knee osteoarthritis and concluded that APS appears safe and can reduce pain and/or improve function, while emphasizing the need for larger randomized trials with active comparators and long-term follow-up (Gupta, 2024). Across these studies, APS was consistently prepared from leukocyte-rich PRP using the nSTRIDE system and delivered as a single intra-articular injection in patients with mild to moderate radiographic knee osteoarthritis (Gupta, 2024).​

In an open-label safety study, King et al. (King et al., 2016) treated 11 patients with knee osteoarthritis using a single APS injection and found no serious adverse events, with early and sustained improvements in WOMAC pain scores over 6 months; importantly, higher white blood cell counts and higher IL-1RA:IL-1β ratios within the APS product correlated with greater pain reduction, suggesting potential biomarkers of response (King et al., 2016). In a separate pilot trial, van Drumpt et al. (van Drumpt et al., 2016) also administered a single APS injection to 11 patients with Kellgren–Lawrence grade II–III knee osteoarthritis and reported a favorable safety profile and significant improvements in WOMAC composite, pain, stiffness, and function subscales from 2 weeks to 6 months, with benefits maintained in most patients at 18-month follow-up (van Drumpt et al., 2016) (Table 1).

**TABLE 1:** Clinical studies of autologous protein solution in osteoarthritis and upper-limb conditions.

Study (first author, year)	Joint/condition	Design and setting	Sample size/key inclusion	Comparator	Follow-up	Main clinical outcomes	Study limitations
(King et al., 2016)	Knee OA	Open-label safety and feasibility study	11 knees with symptomatic knee OA	None (single-arm)	6 months	Single APS injection; no serious AEs; early and sustained improvement in WOMAC pain; higher WBC and IL-1RA:IL-1β ratio in APS associated with greater pain reduction	Very small, uncontrolled, single-arm; short follow-up
(van Drumpt et al., 2016)	Knee OA (KL II–III)	Prospective pilot study	11 patients with KL II–III knee OA	None (single-arm)	6–18 months	Single APS injection; favorable safety; significant improvement in WOMAC total, pain, stiffness and function from 2 weeks to 6 months; benefits maintained in most patients at 18 months	Very small, uncontrolled, single-arm
(Kon et al., 2018)	Knee OA (KL II–III)	Multicenter double-blind RCT	46 unilateral knee OA (APS vs. saline)	Saline intra-articular	12 months	Both groups improved early; at 12 months the APS group had greater improvement in WOMAC pain, some SF-36 domains, and MRI bone marrow lesion/osteophyte scores vs. saline	Moderate sample; single injection only; industry-sponsored
(Kon et al., 2020)	Knee OA (extension of PROGRESS II)	Prospective extension of RCT cohort	Subset of original RCT cohort	Historical saline arm/crossover	36 months	APS recipients maintained significant improvement in WOMAC, KOOS and VAS at 3 years; crossover patients who received APS after saline also improved, with better results in joints without full-thickness cartilage loss	Non-randomized crossover comparison; attrition over 3 years
(Kuwasawa et al., 2023)	Knee OA (KL II–IV)	Retrospective real-world cohort	228 knees enrolled; 148 knees (67%) completed 12-month follow-up	None (single-arm)	12 months	KOOS subscales improved significantly; responder rate 55% overall (58% KL II, 57% KL III, 47% KL IV), lower in KL IV, suggesting reduced efficacy in end-stage OA.	Retrospective; 33% loss to follow-up; no control group
(Kaneko et al., 2026)	Knee OA	Retrospective cohort comparing patients ≥80 vs. < 80 years old	41 patients (44 knees); single APS injection, July 2021–August 2023	Age-stratified comparison (no untreated control)	1 and 6 months	Efficacy (KOOS/KSS improvement) was preserved in patients ≥80 years and comparable to younger patients, supporting APS as an option even in advanced age	Small sample after age stratification; retrospective; short (6-month) follow-up; no placebo arm
(Ross et al., 2024)	Knee OA	Randomized, saline-controlled trial	Adults with symptomatic knee OA	Saline intra-articular	12 months	No significant differences in KOOS or WOMAC between APS and saline over 1 year; pain at 12 months was worse in the APS arm, underscoring uncertainty about efficacy	Single-center; reasons for discrepancy with other RCTs unclear
(Woods et al., 2024)	Shoulder, subacromial impingement	Feasibility randomized trial (APS vs. steroid)	50 patients with subacromial pain	Corticosteroid injection	6 months	APS and steroid injections were both safe with no serious AEs; both groups improved in pain and function; study confirmed feasibility of blinded APS trials but was not powered for definitive efficacy comparisons	Feasibility-level only; not powered for efficacy; single shoulder study identified in the field

In a multicenter, double-blind randomized trial, Kon et al. (Kon et al., 2018) compared a single APS injection with saline in 46 patients with unilateral knee osteoarthritis (KL II–III) and observed similar early improvements in both groups, but by 12 months the APS group showed significantly greater improvement in WOMAC pain, selected SF-36 subscales, and bone marrow lesion and osteophyte scores on MRI, confirming symptomatic benefit and a signal of structural modification versus saline (Kon et al., 2018). In a 3-year extension study, Kon et al. (Kon et al., 2020) followed the same cohort and reported that APS recipients maintained significant improvements in WOMAC, KOOS, and VAS scores at 36 months, with crossover patients who received APS after initial saline also demonstrating meaningful clinical gains, particularly in those without full-thickness cartilage loss at baseline (Kon et al., 2020). In routine clinical practice, Kuwasawa et al. (Kuwasawa et al., 2023) retrospectively evaluated 220 knees treated with APS and found that KOOS subscales improved significantly at 12 months in 55% of patients overall, but responder rates and durability were clearly lower in severe KL grade IV disease than in KL II-III, indicating that APS is more effective in mild-to-moderate than in end-stage osteoarthritis (Kuwasawa et al., 2023).

More recently, an independent randomized, saline-controlled study reported by other investigators found no significant between-group differences in KOOS or WOMAC scores over 1 year and even noted worse pain at 12 months in the APS arm compared with saline, highlighting ongoing uncertainty about the true magnitude and consistency of clinical benefit (Ross et al., 2024). The reasons for this discrepancy remain unclear but may include differences in patient selection, APS preparation protocols, or post-injection inflammatory responses. Together, these data suggest that while APS is generally safe and can provide clinically relevant pain and function improvement in selected knee osteoarthritis populations, its efficacy is not uniformly superior to placebo or standard injectables across all trials, and effect sizes appear attenuated in advanced disease.

For upper-limb and shoulder conditions, Gupta and Maffulli reviewed autologous peripheral blood-derived orthobiologics and identified only a single APS study among six eligible clinical trials, indicating how sparse the APS evidence base is outside the knee (Gupta and Maffulli, 2025). In that feasibility randomized study, Woods et al. (Woods et al., 2024) compared a single subacromial APS injection with a corticosteroid injection in 50 patients with subacromial impingement syndrome and showed that recruitment and retention in a blinded trial were feasible, both treatments were safe with no serious adverse events, and both groups experienced improvements in pain and function scores over 6 months, although the small sample and design precluded definitive efficacy comparisons (Woods et al., 2024). Overall, the shoulder literature supports APS as a safe, potentially useful option for subacromial pain within a broader family of orthobiologics, but robust evidence of superiority over corticosteroids or PRP is lacking.​

### Evidence map

The evidence underlying the three components of the proposed pathway—arthroscopic elbow release, APS, and 3D-printed digital orthopedics—differs substantially in directness, study design, and certainty. To make these differences explicit and to avoid conflating indirect or preclinical data with elbow-specific clinical evidence, Table 2 summarizes the level of evidence available for each component, ranging from direct elbow-specific clinical data to purely conceptual extrapolation. Arthroscopic elbow release is supported by elbow-specific case series, a narrative review, and a pediatric systematic review and meta-analysis, representing the most direct and clinically applicable body of evidence in this review. In contrast, APS is supported by randomized and observational clinical data only in the knee and, to a lesser extent, the shoulder, together with preclinical mechanistic and animal studies; no elbow-specific clinical data exist for APS at any level. 3D-printed planning tools and orthoses are supported mainly by technical feasibility studies, case reports, and small case series around the elbow, without randomized comparisons against conventional techniques or braces. The combined pathway integrating all three components has not been evaluated in any clinical study and remains a conceptual extrapolation. This hierarchy should be kept in mind throughout the remainder of this review: statements regarding the biologic rationale or anticipated benefit of APS in the elbow are hypotheses derived from indirect and preclinical evidence, not established clinical effects.

**TABLE 2:** Evidence hierarchy for the components of the proposed integrated pathway.

Pathway component	Directness of evidence		Study designs available			Certainty of evidence		Key limitations
Arthroscopic elbow release (mechanical correction)	Direct, elbow-specific clinical evidence	Case series (adult and pediatric), narrative review, pediatric systematic review/meta-analysis	Low-to-moderate (observational; no RCTs in adults)	Selection bias, single-surgeon/single-center series, heterogeneous etiology and severity, incomplete adverse-event reporting				
APS in osteoarthritis — knee	Indirect clinical evidence (different joint)		Open-label safety studies, pilot trials, multicenter double-blind RCT with extension, large retrospective cohort	Moderate, but inconsistent (one RCT shows benefit, one shows no benefit/worse pain)		Not elbow-specific; efficacy attenuated in advanced disease; heterogeneous preparation protocols across studies		
APS in shoulder (subacromial)	Indirect clinical evidence (different joint)		Single feasibility RCT vs. corticosteroid			Low (feasibility-level, not powered for efficacy)		Small sample; only one study identified in a systematic review of upper-limb orthobiologics
APS — mechanistic/preclinical	Preclinical evidence		Cartilage explant models, human chondrocyte culture, rodent meniscal-tear osteoarthritis model			Low (mechanistic plausibility only)		No elbow tissue or elbow-joint model tested; translation to human elbow joint unconfirmed
APS in the elbow specifically	No evidence identified		None			Not applicable		Complete absence of elbow-specific data at any level (clinical, preclinical, or case-based)
3D-printed planning models/patient-specific instruments	Technical feasibility evidence		Case report, case series combined with narrative review			Low (no comparative or randomized data)		Small numbers; outcomes largely descriptive (fidelity to intraoperative findings, correction accuracy) rather than patient-centered
3D-printed orthoses/bracing	Technical feasibility evidence		Case report with 1-year follow-up; design/development study without clinical outcomes			Low (no comparative or randomized data)		No trials against conventional thermoplastic or commercial braces; benefit of customization unproven
			Combined pathways (APS + arthroscopic release + 3D-printed devices)		Conceptual extrapolation only	None	Not applicable	Never tested clinically in any combination for the elbow; entirely hypothesis-generating

### Arthroscopic elbow release as a joint-preserving platform

In a landmark series from the Hand and Upper Limb Centre in Canada, Nguyen et al. (Nguyen et al., 2006) evaluated 22 patients undergoing arthroscopic capsular release for elbow contracture with a mean follow-up of 25 months and reported that mean flexion improved from 122° to 141°, mean extension from 38° to 19°, yielding an average arc gain of 38°, with no instability, no major neurovascular complications, and high satisfaction reflected by an ASES-elbow score of 31/36 and minimal DASH impairment, and they concluded that arthroscopic debridement and capsulectomy is an effective treatment for contracted elbows, with outcomes comparable to those reported for open release (Nguyen et al., 2006).​ Building on this, Beck et al. (Beck et al., 2022) analyzed 52 consecutive arthroscopic elbow contracture releases performed by a single surgeon between 2003 and 2015 and demonstrated that in post-traumatic elbows, the flexion-extension arc improved by an average of 63° and pronosupination by 38°, whereas degenerative cases gained 29° in flexion-extension and 13° in forearm rotation, with mean postoperative DASH scores of 17.5 for post-traumatic and 12.8 for degenerative cases. In their series, half of the elbows had severe contractures, yet complication rates remained low, leading the authors to advocate arthroscopic contracture release as an effective option for both post-traumatic and degenerative stiffness and to suggest a staged approach when combined flexion and pronosupination contractures are present (Beck et al., 2022) (Table 3).

**TABLE 3:** Key clinical evidence on arthroscopic elbow contracture release.

Study (first author, year)	Population/indication	n	Design and follow-up	Change in ROM	Key PROs	Complications/comments	Limitations
(Nguyen et al., 2006)	Adults with elbow contracture (post-traumatic and other)	22	Retrospective case series; mean 25 months	Flexion 122°→141°; extension 38°→19°; arc gain ≈38°	ASES-elbow 31/36; minimal DASH impairment; high satisfaction	No instability or major neurovascular injury; comparable to open release	Retrospective, single-center, no comparator
(Beck et al., 2022)	Post-traumatic and degenerative elbow stiffness	52	Retrospective single-surgeon series	Post-traumatic: arc gain 63°; degenerative: arc gain 29°	DASH 17.5 (post-traumatic), 12.8 (degenerative)	Half had severe contracture; low complication rate	Single-surgeon; retrospective; no control group
(Ghayyad et al., 2023)	Post-traumatic elbow stiffness (adults)	32	Prospective case series; short- to mid-term	Arc increased 74°→110° (mean gain 36°)	Significant ASES and MEPI improvement	Transient ulnar nerve paresthesia and superficial infection	Single-center, uncontrolled, short follow-up
(Koukos et al., 2025)	Post-traumatic elbow stiffness, adolescents	—	Narrative review	Comparable to open arthrolysis	Return-to-sport >80%	Complication rate <5% in pediatric/adolescent cohorts	Narrative (non-systematic) review; pediatric population only
(Khorram et al., 2026)	Children/adolescents, open vs. arthroscopic arthrolysis	11 studies pooled	Systematic review and meta-analysis	Open arthrolysis significantly improved ROM; arthroscopic arthrolysis showed a non-significant gain	Low, comparable complication rates between techniques	Findings favor open arthrolysis in severe cases; authors call for larger prospective studies to refine arthroscopic indications	Pediatric-only; open technique outperformed arthroscopic — does not support equivalence claimed in earlier drafts
(Santacaterina et al., 2025)	Adults with post-traumatic elbow stiffness	12 RCTs	Systematic review and meta-analysis of RCTs	Open arthrolysis + early structured rehabilitation significantly improved long-term ROM vs. standard care	—	Certainty of evidence rated low (GRADE); conservative treatment appropriate first-line for mild-moderate cases	RCT base dominated by open technique; arthroscopic-specific RCT data still lacking
(Lanzerath et al., 2023)	Adults, surgical arthrolysis, open vs. arthroscopic	27 studies; 1,666 patients	Systematic review	Comparable good-to-excellent functional outcomes between techniques	—	Revision: 1.6% (arthroscopic) vs. 6.3% (open); complications without revision: 9.1% vs. 18.1%	All included studies observational; no RCT comparing techniques head-to-head

In a more recent prospective case series, Ghayyad and colleagues (Ghayyad et al., 2023) reported on arthroscopic capsular release specifically for post-traumatic elbow stiffness and documented an increase in flexion-extension arc from 74° to 110° (mean gain 36°), with significant improvements in ASES and Mayo Elbow Performance Index scores and a low complication rate limited mainly to transient ulnar nerve paresthesia and superficial infection. The authors emphasized that all patients gained some motion in the previously restricted direction without loss of motion elsewhere, supporting arthroscopic arthrolysis as a safe and reliable option for post-traumatic stiff elbows when performed in appropriately selected cases (Ghayyad et al., 2023).​ Furthermore, Koukos et al. (Koukos et al., 2025) conducted a narrative review of post-traumatic elbow stiffness in adolescents and highlighted that arthroscopic arthrolysis can provide comparable range-of-motion gains to open arthrolysis, with advantages in visualization, infection risk, and return to activities, especially in the absence of severe deformity or extensive heterotopic ossification. They noted that, in experienced hands, indications for arthroscopic arthrolysis can be expanded even in the presence of heterotopic bone or prior surgery, and that return-to-sport rates above 80% and complication rates below 5% have been reported in pediatric and adolescent cohorts (Koukos et al., 2025). Complementing this, a recent systematic review and meta-analysis on pediatric post-traumatic elbow stiffness, pooling 11 studies, found that open arthrolysis produced significantly greater gains in range of motion than arthroscopic arthrolysis, which showed only a non-significant improvement in this pooled pediatric analysis; complication rates, however, were low and comparable between the two techniques (Khorram et al., 2026). This finding tempers rather than reinforces claims of equivalence between open and arthroscopic release, at least in the pediatric population, and, together with the RCT-level data discussed below, underscores that the comparative efficacy of arthroscopic versus open arthrolysis remains incompletely resolved even where safety appears similar.

However, most of the adult evidence summarized above is observational, and the certainty of this evidence is limited. In the only systematic review and meta-analysis of randomized controlled trials specifically addressing post-traumatic elbow stiffness, Santacaterina et al. (Santacaterina et al., 2025) identified twelve RCTs evaluating surgical and conservative interventions and found that open arthrolysis combined with early structured rehabilitation significantly improved long-term range of motion compared with standard postoperative care, but rated the overall certainty of this evidence as low because of methodological limitations across the included trials; the authors concluded that severe or conservative-treatment-refractory cases benefit most from surgical release, while conservative treatment remains appropriate first-line management for mild-to-moderate stiffness (Santacaterina et al., 2025). Notably, this RCT-level evidence base is dominated by open rather than arthroscopic techniques, underscoring that high-certainty comparative data specifically for arthroscopic release remain scarce.

Direct comparisons between open and arthroscopic techniques also remain largely observational. In a systematic review of 27 studies and 1,666 adult patients, arthroscopic arthrolysis was associated with lower reoperation and complication rates than open arthrolysis (revision: 1.6% vs. 6.3%; complications not requiring revision: 9.1% vs. 18.1%), while both approaches achieved comparable rates of good-to-excellent functional outcomes (Lanzerath et al., 2023). However, arthroscopic release carries narrower indications and a recognized learning curve, and is generally reserved for patients with primarily intrinsic or capsular contracture, limited heterotopic ossification, and no major bony deformity; open or combined arthroscopic-open approaches are typically preferred for extrinsic or mixed contractures, extensive heterotopic ossification, significant bony malunion, or revision settings after prior surgery (Lanzerath et al., 2023). Despite these favorable outcomes, neurovascular complications remain the most serious potential risk. The ulnar nerve is especially vulnerable during posterior portal placement and capsular resection. Preoperative ulnar nerve symptoms, prior ulnar nerve transposition, or significant cubital tunnel compression are relative contraindications to standard arthroscopic release, and may warrant concurrent ulnar nerve decompression or transposition.

Taken together, these studies indicate that arthroscopic elbow release can achieve meaningful gains in motion and patient-reported outcomes with a generally favorable complication profile in appropriately selected adult and adolescent patients, but the supporting evidence is predominantly observational, heterogeneous in follow-up duration and outcome reporting, and subject to selection and surgeon-experience bias; the sole RCT-based synthesis available rates the certainty of this evidence as low. This body of evidence positions arthroscopic arthrolysis as a robust mechanical platform onto which biologic adjuncts such as Autologous Protein Solution and technology enabled solutions such as 3D printed planning and orthoses can potentially be layered to enhance joint preservation and long-term outcomes, although this integration itself remains untested (Table 2).

## D-printed digital orthopedics around the elbow

### Patient-specific models and instruments

In a case report on complex post-traumatic elbow stiffness, Grunert et al. (Grunert et al., 2023) used CT-based segmentation and Multi Jet Fusion 3D printing to create a patient-specific, sterilizable elbow model, on which they simulated osteophyte resection before surgery and then replicated the plan intra-operatively. The authors reported that the 3D model substantially improved understanding of the three-dimensional pathology, facilitated accurate preoperative resection planning, and showed a high degree of fidelity to the intra-operative findings, allowing them to avoid residual bony impingement and enhance the quality of arthrolysis (Grunert et al., 2023).​

In a combined case series and narrative review, Roth et al. (Roth et al., 2026) described five complex elbow deformities, including cubitus varus, dorsal radial head subluxation, translational distal humeral deformity, cubitus valgus, and radial head dislocation in hereditary multiple exostoses treated using 3D-based planning and patient-specific instruments (PSIs) developed in collaboration with medical engineers. Across their own cases and 28 additional studies identified in the literature, they showed that 3D-planned osteotomies with PSIs achieve high correction accuracy, particularly for cubitus varus, and in comparative studies often outperform conventional techniques in terms of alignment while reducing operative time and fluoroscopy exposure (Roth et al., 2026). They emphasized that CT-based 3D reconstruction, mirroring of the contralateral limb, virtual osteotomy planning, and PSI-guided cuts are especially useful when small angular or translational errors can have large functional consequences, as in distal humeral malunions and chronic Monteggia-like radial head problems (Roth et al., 2026). Most reported applications to date are open corrective osteotomies for fixed bony deformity rather than arthroscopic procedures for capsular stiffness. Extending this digital workflow—segmentation, virtual planning, and manufacture of patient-specific cutting or drilling guides—to the bony components of elbow arthrolysis or combined arthroscopic–mini-open releases remains a conceptual extrapolation that has not yet been reported in the literature (Table 4).

**TABLE 4:** Key 3D-printed digital orthopedic applications involving the elbow.

Study (first author, year)	Type of 3D application	Indication/setting	Surgical/rehab context	Main outcomes	Relevance to proposed pathway/Limitations
(Grunert et al., 2023)	Patient-specific 3D-printed anatomical model	Complex post-traumatic elbow stiffness with osteophytes	Preoperative planning; arthrolysis	CT-based model allowed simulation of osteophyte resection; high fidelity to intraoperative findings	Directly relevant; single case report
(Roth et al., 2026)	3D-based planning and patient-specific instruments	Complex elbow deformities	Open corrective osteotomies guided by PSIs	High accuracy of deformity correction; reduced operative time/fluoroscopy vs. conventional	Relevant to bony correction; mostly open osteotomy, not arthroscopic capsular release
(Huszar et al., 2024)	Custom 3D-printed hinged elbow splint	Postoperative brace, terrible triad with instability	Post-traumatic reconstruction; early controlled motion	ROM (−24°–138°), low DASH, high ASES/QUEST	Directly relevant; single case report, no comparator
(Cavalcanti, 2023)	Conceptual 3D-printed static-progressive orthosis	Elbow rigidity/stiffness (design study)	Rehabilitation device design	Design-stage device; no clinical outcomes yet	Directly relevant; design study only, not yet clinically tested
(Pan et al., 2025)	Custom 3D-printed elbow prosthesis	Salvage arthroplasty, massive distal humeral bone loss	Revision/salvage arthroplasty	Feasible, satisfactory short-term function in bone loss unsuitable for standard implants	Retained only as a broader additive-manufacturing feasibility example — not direct support for the joint-preserving pathway, since this is joint-replacing surgery

### D-printed orthoses and fixation devices

In a detailed case report, Huszar et al. (Huszar et al., 2024) designed a custom 3D-printed elbow splint for postoperative rehabilitation of a terrible triad elbow injury in a patient with residual instability after ligament repair. Using a handheld 3D scanner, they captured the limb geometry in 90° flexion and full pronation, created a two-piece hinged brace in CAD software, and printed it with a combination of fused deposition modeling and masked stereolithography, allowing adjustable extension limits while maintaining pronation and close conformity to the patient’s arm. Worn continuously from week 2 to week 8 with progressive weekly increases in permitted extension, the splint enabled early controlled mobilization and, at 1 year, the patient achieved an arc from −24° extension to 138° flexion, near-normal pronation–supination, low DASH (14.16), high ASES (92), and a high QUEST satisfaction score (56/60) (Huszar et al., 2024).​

On the other hand, Cavalcanti et al. (Cavalcanti, 2023) developed a digital model of an articulated elbow orthosis specifically aimed at rehabilitation of joint rigidity, progressing through “Discover, Define, Develop, Deliver” phases with input from patients, orthopaedic surgeons, physiotherapists, and 3D-printing specialists. Their proposed device is a static-progressive articulated orthosis, customized via 3D scanning and CAD, printed with fused deposition modeling, and equipped with hinges and hooks for elastic bands or springs to deliver controlled flexion and extension loads, with the explicit goal of maintaining or increasing range of motion while respecting soft-tissue tolerance. Although their work is largely developmental and not yet supported by a clinical outcome series, it demonstrates how 3D-printed orthoses can operationalize established principles of static-progressive stretch in a highly customizable, lower-cost format (Cavalcanti, 2023).​

Thus, the clinical experience of Huszar and the design work of Cavalcanti illustrate two complementary roles for 3D-printed elbow orthoses (Fan et al., 2025): enabling early but protected motion in unstable or complex reconstructions, and (Akhtar et al., 2021) delivering tailored static-progressive stretching for chronic stiffness. These reports are based, respectively, on a single case and a design study without clinical outcomes, and no comparative trials against conventional thermoplastic or commercial orthoses currently exist; 3D-printed devices should therefore be regarded as a promising but unproven alternative rather than an established improvement over conventional bracing. Within the conceptual framework of the present review, such personalized 3D-printed devices can provide the mechanical environment, controlled motion, optimized fit, and improved comfort, in which biologic adjuncts like APS and surgically restored joint mechanics are most likely to translate into durable functional gains.

### Rehabilitation considerations

Rehabilitation is a central determinant of outcome throughout the management of post-traumatic elbow stiffness, yet the evidence base guiding specific rehabilitation decisions remains limited and heterogeneous. In the only systematic review focused specifically on conservative treatment of post-traumatic elbow stiffness, Piacenza et al. (Piacenza et al., 2024) identified five eligible studies and found that, despite substantial heterogeneity in interventions precluding meta-analysis, large effect sizes were observed when conservative treatment was initiated promptly after the period of immobilization, with proprioceptive neuromuscular facilitation and graded motor imagery performing more favorably than comparator interventions for functional recovery and pain; however, the certainty of evidence for all outcomes was rated low to very low using GRADE methodology, and the authors concluded that no single conservative modality could be recommended with confidence (Piacenza et al., 2024).

#### Conservative management before surgery

Initial treatment of post-traumatic elbow stiffness is nonoperative in most patients, combining structured physiotherapy, active and active-assisted range-of-motion exercises, and splinting; surgical release, including arthroscopic arthrolysis, is generally reserved for patients who fail an adequate trial of conservative management or who have a mechanical block to motion such as heterotopic ossification or malunion (Piacenza et al., 2024).

#### Immediate postoperative rehabilitation

Early initiation of motion after arthroscopic or open release is widely emphasized as important for maintaining the gains achieved intraoperatively and limiting recurrent fibrosis, though the optimal timing, frequency, and intensity of early mobilization protocols have not been established by comparative trials (Verstuyft et al., 2021).

#### Pain and edema management

Effective control of postoperative pain and swelling is a prerequisite for tolerating early active mobilization; cryotherapy, elevation, and multimodal analgesia are commonly used, although elbow-specific comparative data on their impact on final range of motion are lacking.

#### Active versus passive mobilization

Active and active-assisted motion is generally favored over purely passive mobilization or prolonged continuous passive motion, based on the general orthopedic principle that active muscle contraction assists in maintaining gains achieved surgically, though elbow-specific randomized comparisons are absent from the available literature.

#### Static-progressive and dynamic splinting

Splinting plays a central role once a stable range has been achieved, and this is an area where 3D-printed orthoses may offer practical advantages, as illustrated by the custom hinged brace of Huszar et al., which allowed adjustable, progressively increasing extension limits during controlled postoperative mobilization (Huszar et al., 2024), and the static-progressive articulated orthosis conceptualized by Cavalcanti et al. (Cavalcanti, 2023). However, no trial has directly compared 3D-printed with conventional thermoplastic or commercial static-progressive or dynamic splints, and the incremental benefit of personalization remains unproven.

#### Strengthening, functional recovery, and return to work or sport

Once range of motion has stabilized, progressive strengthening is typically introduced to support functional use of the limb; in surgical case series, return-to-sport rates above 80% have been reported in adolescent cohorts after arthroscopic release (Koukos et al., 2025), but comparable functional and return-to-work data in adults are sparse and inconsistently reported across studies.

#### Criteria for progression and referral

In the absence of validated elbow-specific rehabilitation protocols, progression through phases of mobilization, strengthening, and functional return is generally guided by clinical milestones (pain control, absence of effusion, achievement of a stable range of motion) and surgeon judgment rather than by standardized, evidence-based criteria; this represents an important gap for future research (Piacenza et al., 2024).

### Conceptual integration: APS, arthroscopic release, and 3D-Printed digital orthopedics

Drawing on the evidence reviewed above, arthroscopic elbow release can be viewed as the central mechanical intervention, with APS and 3D-printed technologies acting as biologic and structural adjuncts in a joint-preserving pathway. Arthroscopic capsular release and osteophyte debridement, as demonstrated by Nguyen et al. (Nguyen et al., 2006) and Beck et al. (Beck et al., 2022), improve range of motion and patient-reported outcomes with a generally favorable complication profile in selected patients, based predominantly on observational evidence (Table 2), but do not fully address ongoing synovial inflammation or cartilage degeneration in some patients. In parallel, the knee osteoarthritis studies summarized by Gupta and colleagues suggest that intra-articular APS can modulate inflammatory pathways and improve symptoms in selected patients, particularly in mild-to-moderate disease, although recent randomized data remain mixed (Gupta, 2024). Studies of Grunert et al. (Grunert et al., 2023) and Roth et al. (Roth et al., 2026) further show that 3D-printed anatomical models and patient-specific instruments improve understanding of complex elbow deformities and can enhance the precision of bony correction.

Within this context, a conceptual integrated pathway could involve (Fan et al., 2025): CT-based 3D reconstruction and virtual planning for elbows with complex post-traumatic stiffness or deformity, using models or PSIs to optimize arthroscopic and mini-open bony work; (Akhtar et al., 2021); arthroscopic release as the core procedure to restore motion and remove mechanical impingement; and (Vasen et al., 1995) immediate or early intra-articular APS injection to dampen postoperative inflammatory cascades and support cartilage preservation in joints with early degenerative change. We emphasize that the timing, safety, and clinical benefit of APS injection in the immediate or early postoperative arthroscopic setting have not been evaluated in the elbow, or in any other joint following arthroscopic surgery, and this specific sequencing should be regarded as a research hypothesis rather than a practical protocol for current clinical use. Postoperatively, custom 3D-printed orthoses such as the hinged brace described by Huszar et al. (Huszar et al., 2024) or the static-progressive orthosis conceptualized by Cavalcanti et al. (Cavalcanti, 2023) could provide patient-specific control of motion, balancing the need for early mobilization, emphasized in postoperative elbow rehabilitation reviews, against protection of repaired soft tissues and biologically vulnerable cartilage. Importantly, this pathway remains hypothetical for the elbow. Its clinical value requires prospective validation, particularly regarding the added benefit of APS in this specific surgical context. Nonetheless, it illustrates how mechanical, biologic, and digital-orthopedic elements might be combined in a coherent framework (Figure 2).

**FIGURE 2:**
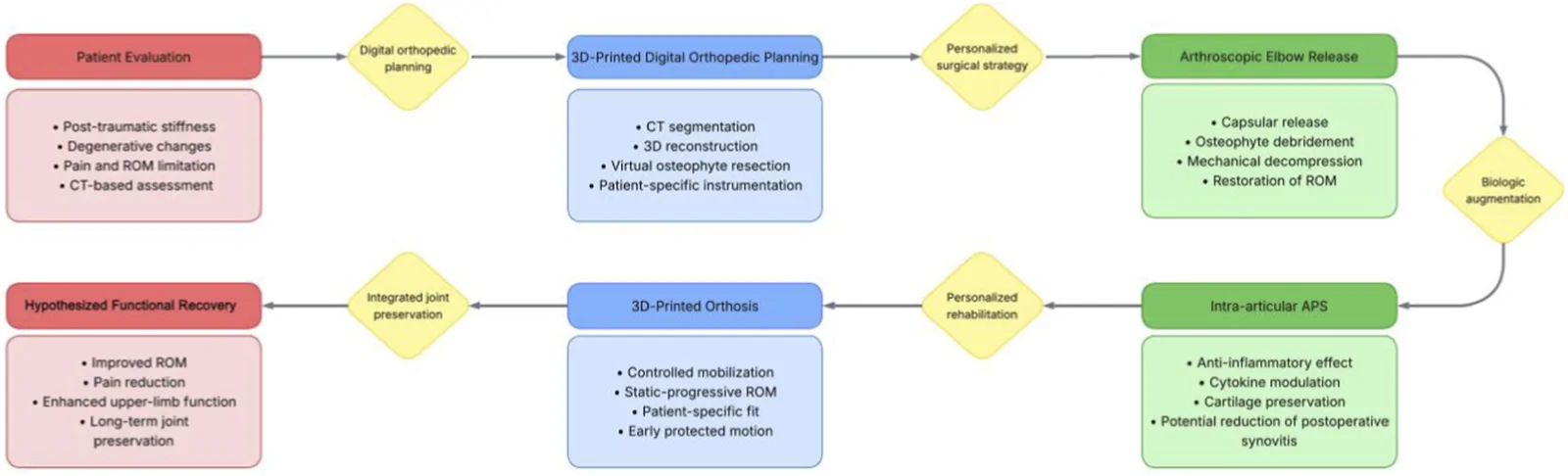
Comprehensive digital and biological pathway for arthroscopic management of elbow stiffness, integrating 3D-printed planning, arthroscopic release, autologous protein solution, and personalized 3D-printed bracing.

## Patient selection and clinical applicability

The evidence reviewed above spans a heterogeneous range of post-traumatic and degenerative elbow conditions, and the hypothetical integrated pathway proposed in this review is not intended to apply uniformly across this spectrum. Based on the populations studied in the arthroscopic release literature, the proposed framework is most conceptually applicable to adult and adolescent patients with post-traumatic or mild-to-moderate degenerative elbow stiffness in whom the predominant pathology is intrinsic or capsular contracture with a mechanically treatable component and early-stage cartilage degeneration, rather than advanced joint destruction (Beck et al., 2022; Koukos et al., 2025; Lanzerath et al., 2023).

Factors that should be considered in defining candidacy for such an approach, and that are inconsistently reported across the studies discussed in this review, include patient age; cause and duration of stiffness; the relative contribution of intrinsic (articular/capsular), extrinsic (soft-tissue), or mixed pathology; degree and radiographic severity of cartilage damage or osteoarthritis; presence of elbow instability; extent of heterotopic ossification; history of previous elbow surgery; presence of ulnar neuropathy; and prior failure of adequate conservative treatment (Ravalli et al., 2019; King et al., 2016; Lanzerath et al., 2023).

Conversely, this framework is unlikely to be generalizable to patients with advanced joint destruction, major bony deformity or malunion, chronic instability, extensive heterotopic ossification, or severe ulnar neuropathy, all of which typically require open or combined approaches and fall outside the populations in whom arthroscopic release, APS, and 3D-printed bracing have been studied, even indirectly (Beck et al., 2022; Lanzerath et al., 2023). Contraindications to arthroscopy itself—including preoperative ulnar nerve symptoms, prior ulnar nerve transposition, or significant cubital tunnel compression without concurrent nerve management—would also apply to any integrated pathway built on an arthroscopic platform. Defining these boundaries explicitly is essential given the conceptual, non-validated nature of the proposed pathway.

### Safety considerations

Because the integrated pathway proposed in this review has not been clinically tested, its overall safety profile is unknown and must be inferred from the safety data available for each component individually.

#### APS-related risks

Across knee osteoarthritis trials, APS injections have generally been well tolerated, with adverse events limited mainly to mild, self-limited injection-site discomfort, transient joint pain, and procedural nausea (van Drumpt et al., 2016). However, moderate temporary post-injection flares have been reported and should be anticipated, even though they do not appear to affect longer-term clinical outcomes (Van Genechten et al., 2021). Whether the same safety profile applies to the elbow, a smaller joint with a thinner soft-tissue envelope, has not been evaluated.

#### Timing relative to arthroscopy

The sequence proposed in this review—arthroscopic release followed by immediate or early intra-articular APS injection—has no direct safety evidence in the elbow or in any other joint following arthroscopic surgery. Postoperative hemarthrosis and reactive synovitis are expected after arthroscopic arthrolysis, and it is unknown how these processes interact with an intra-articularly injected biologic product, whether with respect to dilution, altered local inflammatory response, or infection risk. Until such data exist, this sequence should be treated strictly as a research hypothesis and not applied as a practical clinical protocol.

#### Neurovascular risk of arthroscopy

As noted above, the ulnar nerve is the structure most at risk during elbow arthroscopy, particularly during posterior portal placement and capsular resection; recognized risk factors include preoperative ulnar nerve symptoms and prior ulnar nerve surgery, and surgeon experience is inversely associated with complication rates (Lanzerath et al., 2023).

#### Orthosis-related risks

Custom 3D-printed orthoses introduce considerations distinct from conventional bracing, including possible skin pressure points, hygiene and cleaning of complex printed geometries, heat retention or discomfort from certain print materials, and mechanical failure of printed hinges or fasteners under repeated cyclical loading; none of the case reports or design studies reviewed here (Huszar et al., 2024; Cavalcanti, 2023) formally assessed these outcomes, and they should be addressed prospectively as this technology matures.

#### Imaging, data, and manufacturing considerations

Workflows that rely on CT-based segmentation for patient-specific models or instruments (Roth et al., 2026; Grunert et al., 2023) expose patients to ionizing radiation beyond what standard elbow imaging requires, and the marginal clinical benefit of this additional imaging has not been formally weighed against its radiation burden. In addition, digital anatomical data must be stored and transferred securely, and printed devices intended for surgical planning or patient contact require appropriate quality control and, where relevant, sterilization processes consistent with local regulatory requirements; these logistical and regulatory dimensions are discussed further in the following section.

### Practical and regulatory considerations

Beyond rehabilitation protocols themselves, enhanced recovery after surgery (ERAS) pathways, such as the randomized trial by Cui et al. (Cui et al., 2019) in open elbow arthrolysis, have shown that multimodal perioperative protocols can reduce early postoperative pain and improve range of motion without increasing complications, supporting a systematic, protocol-driven approach to postoperative care (Cui et al., 2019). Such structured perioperative pathways provide a natural framework into which APS injections and 3D-printed orthoses could eventually be integrated, but their use for this purpose would require clear, prospectively defined protocols for timing, dosing, and rehabilitation milestones, none of which currently exist for the elbow.

Moreover, Gupta’s review (Gupta, 2024) noted that APS preparation requires dedicated kits and processing infrastructure, with costs substantially higher than standard intra-articular injections such as corticosteroids or hyaluronic acid, and that reimbursement pathways vary greatly between health systems. In many jurisdictions, APS use in joints other than the knee, and particularly in combination with elbow arthroscopy, would be off-label, necessitating careful informed consent, ethical oversight, and possibly institutional review board approval for formal clinical studies. Successful integration of APS and 3D-printed digital orthopedics into elbow arthroscopic pathways will therefore require not only clinical protocols but also solutions to manufacturing logistics, cost allocation, reimbursement, and regulatory compliance.

## Future directions

Future research should first validate the proposed integrated pathway in well-defined clinical cohorts. Pilot randomized or controlled studies comparing arthroscopic contracture release with versus without intra-articular APS in patients with post-traumatic elbow stiffness and early osteoarthritic changes could use standardized outcomes such as range of motion, Mayo Elbow Performance Score, DASH, pain scales, and imaging markers of cartilage status, extrapolating design elements from existing APS trials in knee osteoarthritis. Given the complete absence of elbow-specific APS data, an initial safety and feasibility study would be essential before any efficacy trial. Within such studies, incorporating custom 3D-printed braces similar to those described by Huszar et al. (Huszar et al., 2024) would allow early but controlled mobilization in both arms, helping to isolate the biologic effect of APS while simultaneously generating data on adherence, comfort, and functional impact of personalized orthoses. At the same time, translational work is needed to tailor APS protocols to the elbow joint, including investigations of optimal timing relative to arthroscopy, single versus repeated injections, and potential biomarkers such as APS cytokine ratios or baseline cartilage status to identify likely responders, building on mechanistic and clinical insights from the knee. *In vitro* studies using human elbow cartilage explants would be valuable to confirm that APS has similar chondroprotective effects in elbow tissue as observed in knee models. In the digital orthopedics domain, priorities include streamlining CT-based modeling workflows, quantifying the impact of 3D-printed models and patient-specific instruments on operative time and accuracy in elbow procedures, and performing formal cost-effectiveness analyses to determine in which clinical scenarios customization is justified. Randomized trials comparing 3D-printed orthoses to standard commercial braces are needed to validate the assumed benefits of customization. As evidence accumulates, consensus-building efforts similar to existing guidelines for post-traumatic stiff elbow and elbow rehabilitation could incorporate orthobiologic and 3D-printed components, moving this concept from hypothesis to structured clinical pathways.

## Conclusion

Post-traumatic and degenerative elbow stiffness remains a challenging clinical problem, as even small losses of motion can cause disproportionate disability. Arthroscopic elbow release has emerged as an effective joint-preserving technique for restoring motion, with a generally favorable complication profile in appropriately selected patients, although this is supported predominantly by observational rather than high-certainty comparative evidence. In parallel, Autologous Protein Solution has shown biologically plausible mechanisms and encouraging but heterogeneous clinical results in knee osteoarthritis and limited upper-limb indications, while 3D-printed digital orthopedics has demonstrated promising, though largely case-based, advantages for preoperative planning, patient-specific instrumentation, and customized orthoses around the elbow.

No clinical study has combined APS, arthroscopic elbow release, and 3D-printed technologies for the elbow, and elbow-specific clinical evidence for APS is entirely absent; the integrated pathway proposed in this review is therefore a hypothesis-generating conceptual framework, not a validated or clinically actionable protocol. Addressing these gaps through carefully designed translational and clinical studies, beginning with elbow-specific safety and feasibility data for APS, will be essential to move this concept from theoretical integration toward evidence-based protocols for managing stiff elbows in a personalized, joint-preserving manner.
